# RUNX1: an emerging therapeutic target for cardiovascular disease

**DOI:** 10.1093/cvr/cvaa034

**Published:** 2020-03-10

**Authors:** Alexandra Riddell, Martin McBride, Thomas Braun, Stuart A Nicklin, Ewan Cameron, Christopher M Loughrey, Tamara P Martin

**Affiliations:** c1 British Heart Foundation Glasgow Cardiovascular Research Centre, Institute of Cardiovascular & Medical Sciences, University of Glasgow, 126 University Place, Glasgow G12 8TA, UK; c2 Max Planck Institute for Heart and Lung Research, Ludwigstr. 43, 61231 Bad Nauheim, Germany; c3 School of Veterinary Medicine, University of Glasgow, Garscube Campus, Glasgow G61 1BD, UK

**Keywords:** Runx1, Myocardial infarction, Adverse cardiac remodelling, Cardiovascular diseases, Heart failure, Excitation–contraction coupling, Calcium

## Abstract

Runt-related transcription factor-1 (RUNX1), also known as acute myeloid leukaemia 1 protein (AML1), is a member of the core-binding factor family of transcription factors which modulate cell proliferation, differentiation, and survival in multiple systems. It is a master-regulator transcription factor, which has been implicated in diverse signalling pathways and cellular mechanisms during normal development and disease. RUNX1 is best characterized for its indispensable role for definitive haematopoiesis and its involvement in haematological malignancies. However, more recently RUNX1 has been identified as a key regulator of adverse cardiac remodelling following myocardial infarction. This review discusses the role RUNX1 plays in the heart and highlights its therapeutic potential as a target to limit the progression of adverse cardiac remodelling and heart failure.

## 1. Introduction

**Figure 1:**
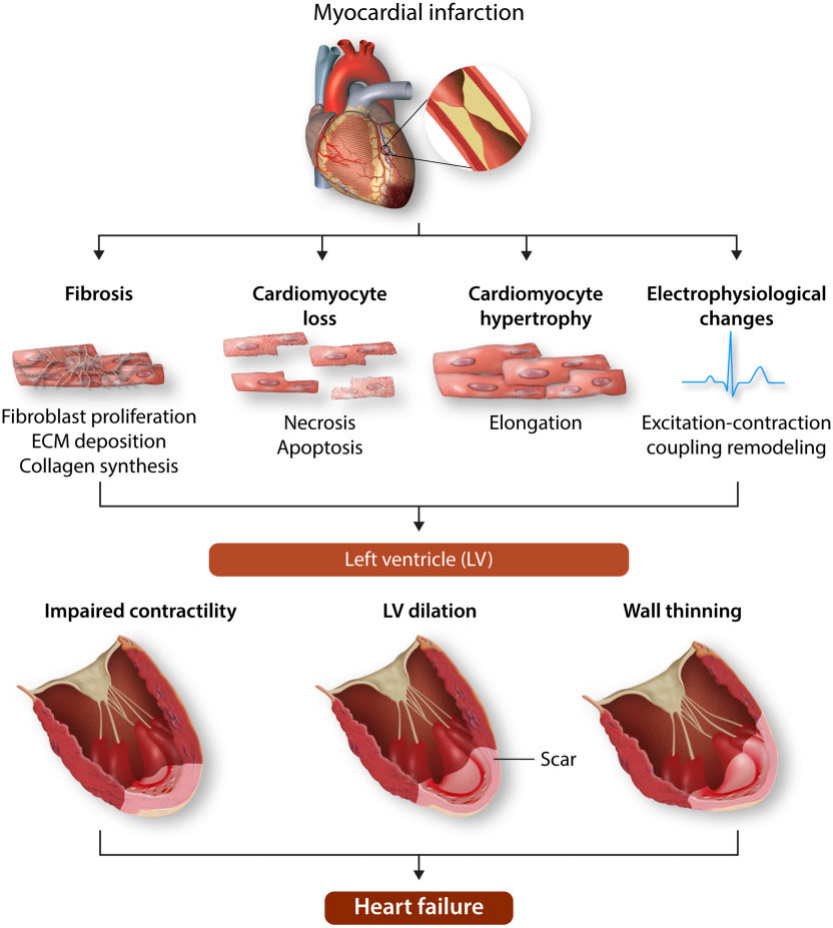
Pathological ventricular remodelling. Schematic showing remodelling of the left ventricle (LV) following myocardial infarction (MI). Following MI, a fibrosous scar forms in the infarcted tissue accompanied by myocyte loss. Adjacent to the infarcted tissue in the region bordering the remote LV, the border-zone region, myocytes thicken and elongate and excitation–contraction coupling becomes impaired. In terms of gross architecture of the LV, the early phase of remodelling is characterized by thinning and elongation of the infarcted zone followed by ventricular dilation, where the LV transitions from an elliptical shape to a spherical shape. Schematic was prepared using Servier Medical Art by Servier under a Creative Commons Attribution 3.0 Unported License.

The healthcare burden and socioeconomic impact of heart failure is increasing on a global scale leading to significant levels of disability, reduced quality of life, and mortality.^1^^,^^2^ Adverse changes in the architecture and function of the heart, collectively referred to as cardiac remodelling, are critical to the development of heart failure. Adverse cardiac remodelling occurs in response to many cardiovascular diseases including acute myocardial infarction (MI), hypertension, arrhythmias, and valve disease. For example, following an MI, adverse cardiac remodelling clinically manifests as left ventricular (LV) wall thinning, impaired contractility, and dilation; progression of which is linked to increased deaths or hospitalizations due to heart failure (*Figure 1*).^3^

At the cellular and molecular level, adverse cardiac remodelling is the result of a complex series of alterations to transcriptional, structural (e.g. hypertrophy), and electrophysiological signalling pathways (*Figure 1*) in cardiomyocytes.^4^ These changes are accompanied by fibroblast proliferation and collagen deposition (fibrosis), vascular smooth muscle cell proliferation, endothelial dysfunction, and inflammation.^4^

Current long**-**term therapy to prevent or reverse adverse remodelling remains inadequate and therefore novel strategies to preserve LV function and limit adverse cardiac remodelling are needed to treat patients with cardiovascular disease and improve prognosis.^5^ This review discusses the structure and function of the master-regulator transcription factor RUNX1. In particular, we discuss its novel and far-reaching role within the cardiovascular system, highlighting it as a target of interest for adverse cardiac remodelling that merits further experimental and clinical investigation.

## 2. RUNX1

The RUNX (runt-related) family of genes encode the α-subunits of a family of transcription factors that orchestrate proliferation, differentiation, and cell survival in multiple lineages. In mammalian species, three α-subunits exist, known as RUNX1, RUNX2, and RUNX3, each with its own distinct spatial-temporal and tissue-specific pattern of expression.^6^^,^^7^ All the RUNX proteins partner with a constitutively expressed β subunit (core-binding factor β; CBFβ), to form a transcriptionally active heterodimer that can either activate or repress target gene expression. Although each RUNX protein interacts with the same target consensus sequence, they display distinct and non-redundant biological functions.^7^

RUNX1 is best characterized for its role as a key transcriptional regulator of haematopoiesis and its involvement in blood malignancies.^8^ Mice with a homozygous knockout of *Runx1* lack definitive haematopoiesis and are unable to survive past an early embryonic stage (days 11.5–12.5) due to severe haemorrhage within the central nervous system (CNS), peritoneum, and pericardium.^9^ In humans, the *RUNX1* gene is one of the most common targets of chromosomal and genetic alterations in acute leukaemia.^10^ Whilst the focus of RUNX1 research has therefore predominately been in the cancer field, accumulating evidence suggests that RUNX1 has more widespread functions in a range of organs and pathologies than previously considered.

## 3. RUNX1 structure and regulation

**Figure 2:**
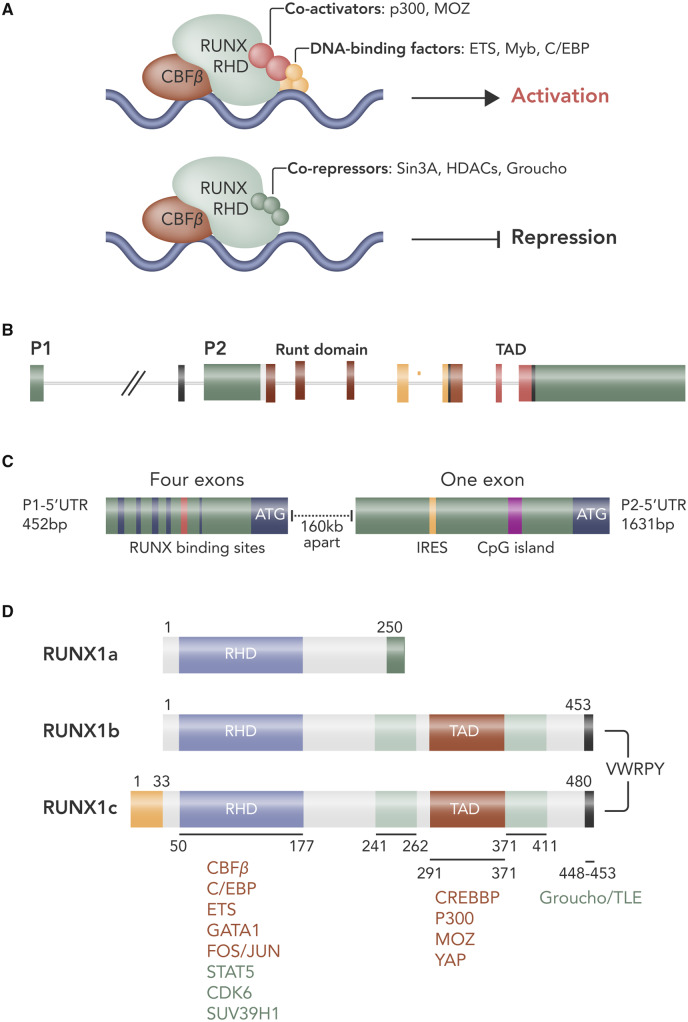
RUNX1 structure and regulation. Illustration of RUNX1 transcriptional regulatory complexes for activation and repression of gene expression (*A*). Schematic depicting RUNX1 gene structure (*B*). Expression of RUNX1 is initiated by two promoters: distal P1 and proximal P2. (*C*) Alternative promoters and gene-splicing results in different 5**ʹ-**UTRs. The P1-5**ʹ-**UTR contains four exons and the P2-5ʹ**-**UTR has a single exon and an internal ribosome entry site (IRES). This results in three major isoforms of RUNX1; 1A and 1B transcribed from P2 and isoform 1C transcribed from P1. (*D*) Schematic of the proteins encoded by the RUNX1 isoforms with the major functional domains marked: runt-homology domain (RHD) and transactivation domain (TAD). Figure prepared using information from various sources.^186–188^

Like the other members of the RUNX family, the RUNX1 protein contains the highly conserved 128 aa region known as the Runt homology (runt) domain which mediates binding to DNA and facilitates interaction with CBFβ.^11^ In its free form, RUNX1 has a relatively poor capacity to bind DNA due to negative regulation from regions at the N- and C-terminal limits of the Runx domain.^12^^,^^13^ On binding to CBFβ, structural rearrangements of the runt domain take place which unmask and stabilize the DNA-binding site resulting in a markedly increased affinity for DNA.^12^^,^^14^ Moreover, whilst the RUNX1 protein itself has a short half-life of approximately 60 min, formation of the RUNX1–CBFβ heterodimer protects RUNX1 from degradation by the ubiquitin ligase complex, increasing its half-life three-fold.^15^ Transcriptional ability is further modulated via protein-interacting domains within the RUNX1 C-terminus including the PY motif (proline-rich peptide that interacts with proteins with a WW domain), and the VWRPY motif [which acts as a docking site for the assembly of the Groucho/Transducin-like enhancer of split (TLE) family of co-repressor proteins].^16^^,^^17^ Via these domains, RUNX1 can form a platform for the assembly of other transcription factors, co-activators, and chromatin modulators to form large transcriptional complexes^16^ (*Figure 2A*). It is likely that the particular combination of mediators recruited to these complexes dictate the profile of the genes targeted and whether they are activated or repressed, leading to both indirect regulation and context-dependent function.^16^

Expression of RUNX1 itself is tightly controlled at the transcriptional, translational, and post-translational level, which also contributes to the strong context specificity of RUNX1 function. The *RUNX1* gene is under the control of a dual promoter system comprising a distal P1 and a proximal P2 promotor and alternative usage of these promoters leads to the generation of mRNA isoforms that differ in their 5ʹ-untranslated region (UTR) and N-terminal coding sequences^18^^,^^19^ (*Figure 2B, C*). The biological significance of the dual promoter system has not been fully elucidated; however, their differential expression during developmental haematopoiesis appears important for cell specification.^20^ Expression of the P2 transcript predominates during haematopoietic stem and progenitor cell (HSC) emergence from the haemogenic endothelium during embryonic development, whereas P1 transcript expression increases as HSCs colonize the liver and becomes the dominant isoform in both liver HSCs and in adult HSCs in the bone marrow.^20^^,^^21^ The differing lengths of the 5ʹ-UTR of the P1 and P2 transcripts may also facilitate their differential expression in distinct cellular contexts by influencing their translation efficiency. The human *RUNX1* P1 transcript has a 5ʹUTR that is 452 base pairs (bp) in length and is translated efficiently; however, the 5ʹ-UTR of the P2 transcript is substantially longer at 1631 bp and forms a secondary structure which appears to hinder its translation *in vitro*.^22^ Whilst the P1-5ʹ-UTR is translated by a cap-dependent mechanism (the most common means of translation direction under basal conditions), translation of P2 transcripts is directed by the presence of an internal ribosomal entry site (IRES), which recruits the ribosome to directly initiate translation thus allowing the canonical, cap-dependent mode of translation initiation to be bypassed.^22^ IRES-mediated translation has been suggested to predominate under conditions of stress, such as hypoxia or apoptosis when cap-dependent translation is diminished.^23^ Messenger RNA isoforms with different translational regulation may allow a shift from the predominant expressed RUNX1 protein isoform and may tailor RUNX function as part of an adaptive response to stress.^7^

Further diversity in the *RUNX1* gene products is augmented by splicing and exon skipping of the P1 and P2 transcripts to yield further isoforms with different functions and expression patterns.^7^^,^^18^ For example, in humans, P1-driven transcription produces the *RUNX1c* isoform, whereas P2 activity yields the *RUNX1a* and *RUNX1b* isoforms.^18^ This gives rise to a range of protein isoforms, ranging in size from 20 to 52 kDa^18^^,^^24^ (*Figure 2D*). RUNX1a is truncated at exon 7 and so has DNA-binding capacity but lacks the C-terminal transcriptional regulatory domain seen in RUNX1b and RUNX1c. It has therefore been suggested that RUNX1a may act as an antagonist to transcriptional activation driven by RUNX1b and RUNX1c.^18^^,^^24^ Indeed, in a murine myeloid cell line, ectopic expression of AML1a (RUNX1a) only or a combination of AML1a and AML1b (RUNX1b) was found to have an opposing effect on the response of the cells to granulocyte colony-stimulating factor (G-CSF). Overexpression of AML1a alone was found to block granulocytic differentiation induced by G-CSF, whereas additional expression of AML1b was found to rescue granulocytic differentiation.^25^

**Figure 3:**
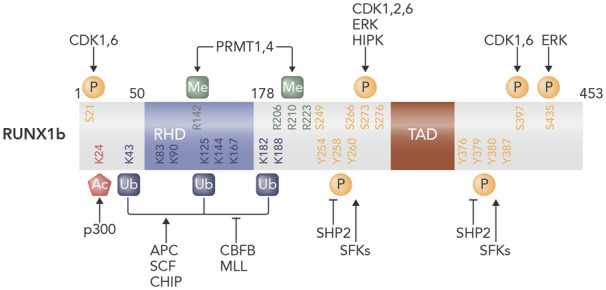
Post-translational modifications of human RUNX1. Schematic depicting post-translational modifications of RUNX1b. Runt**-**homology domain (RHD) is the DNA- and CBFβ-binding domain. Transactivation domain (TAD) is important for transcriptional activation. Numbers refer to amino acid residues from N terminus. Ac, acetylation; APC, anaphase-promoting complex; K, lysine; Me, methylation; P, phosphorylation; R, arginine; S, serine; SCF, Skp1/Cullin/F-box protein complex; T, threonine; Ub, ubiquitination; Y, tyrosine. Figure prepared using information from various sources.^189^^,^^190^

Additional control over RUNX1 stability and function is achieved by a range of post-translational mechanisms including phosphorylation, acetylation, and ubiquitination (*Figure 3*). Acting as scaffolds for multiple protein complexes the outcome of RUNX binding to DNA is heavily influenced by interaction with different activating or repressive co-factors. Post-translational modifications can influence RUNX1 activity, affinity for DNA, and its stability at least in part by directing its binding to other co-factors and in this way provides a means to fine-tune RUNX1 activity in response to external stimuli or cellular events.^26^ For example, phosphorylation was shown to enhance the transactivation activity of RUNX1.^27^ Subsequently, phosphorylation has been shown to reduce interaction with histone deacetylases^28^ and other repressors such as sin3a.^29^ Conversely, phosphorylation (albeit on specific residues) has also been shown to reduce activity.^30^ RUNX1 can be phosphorylated in response to cytokine/growth factor-induced signalling, underlining how transcription factor activity can be modulated by external factors.^30^ Given the importance of post-translational modifications it is not surprising that aberrant phosphorylation of RUNX1 has been linked to disease. FMS-like tyrosine kinase 3 (FLT3) mutations resulting in constitutive activation are common in AML and have been correlated with high levels of RUNX1. A study by Behrens *et al.*^31^ revealed that constitutive FLT3 activation was associated with phosphorylation of RUNX1 residues in the inhibitory domain of RUNX1, resulting in increased expression of downstream oncogenic targets. Thus, it is possible that FLT3 activation may recruit RUNX1 as its oncogenic partner at least in part through modifying its activity.

Other modifications to RUNX1 affect its interaction with regulatory co-factors, for example, methylation can affect RUNX1 partner interactions to both promote^32^ and repress^33^ transcriptional activity. Similarly, p300-mediated acetylation of RUNX1 has been associated with increased DNA binding and transcriptional activity.^34^ Ubiquitination frequently marks proteins, including RUNX1, for degradation by the proteasome but its effects can be more subtle and, in some cases, can result in protein stabilization.^35^

The highly complex regulation of RUNX1 expression is perhaps reflective of a need to tightly control and direct the cellular processes of cellular differentiation, proliferation, and lineage commitment in which RUNX1 is critical. Consequently, dysregulation or altered expression of RUNX1, which is well documented in cancer patients, leads to disrupted cellular function and disease.^10^

## 4. RUNX1 in the developing heart

In the developing embryo, RUNX1 is the most broadly expressed of all the RUNX proteins and is expressed in a range of tissues including the mesenchymal tissue of the heart and in vascular tissue.^6^ The importance of RUNX1 in the development of the vasculature is highlighted in the phenotype observed in *Runx1* KO mice. These mice show a complete lack of definitive haematopoiesis and abnormal vasculature development in many organs.^9^ In the heart, these mice have an underdeveloped coronary plexus and smaller ventricular free wall vessels.^36^ This coincides with changes in heart structure including ventricular septal defects and the development of thin myocardium. In *ex vivo* preparations of explanted ventricle from embryonic stage 11.5, *Runx1* null preparations showed less endothelial sprouting.^36^ The effects on the developing vasculature may be linked to a haematopoietic function; however, a contributing non-haemogenic role of RUNX1, perhaps directly affecting vessel and heart structure cannot be ruled out based on this study alone.^36^^,^^37^

Expression of RUNX1 in the neonatal heart is higher compared with adult heart tissue.^38^ Although the reasons for this are unexplored, it is interesting to note that genes with RUNX1-binding sites within their promoter region are overrepresented in the collection of genes that become methylated during the first week of life.^39^ Promoter methylation is an epigenetic modification which usually leads to gene silencing by blocking the access of transcriptional machinery.^40^ In this setting, increased gene methylation may be important in the maturation process by switching off genes necessary for heart development to support transition to a more adult phenotype.^39^ Of note, methylation of RUNX1 target genes during the 7 days after birth coincides with a loss of the hearts proliferative and regenerative capacity.^39^ This is an intriguing finding given the fact that there is a known link between RUNX1 and the transcriptional co-activators Yap (Yes-associated protein) and TAZ (transcriptional co-activator with PDZ binding motif), which have a regenerative role in the neonatal and adult heart.^41–43^

As RUNX1 has been shown to be involved in female sex development and is a mediator of female hormone signalling, with RUNX1 dysregulation involved in breast, ovarian, and uterine cancers,^44^^,^^45^ it is important to consider that RUNX1 may have differing roles within the female vs. male cardiovascular system.

## 5. RUNX1 expression in cardiac injury

Although expression of RUNX1 in the adult heart is reported to be low, several studies have demonstrated that RUNX1 is increased in the context of cardiac pathology.^46–49^ This was first demonstrated by Gattenlohner *et al.*^48^ in 2003 using human heart tissue autopsies from patients with diagnosed ischaemic cardiomyopathy. Up-regulation of *RUNX1* mRNA in the ischaemic tissue was accompanied by overexpression of the 52 kDa RUNX1 isoform and down-regulation of a 38 kDa isoform predicted to be the RUNX1 AML-1 delta N isoform which has a known dominant-negative function.^48^^,^^50^ In a separate study, RUNX1 was also up-regulated in heart tissue from patients with chronic dilated cardiomyopathy compared with control myocardium.^47^ These findings have been reinforced by rodent models of MI which have shown induction of RUNX1 in the nuclei of cardiomyocytes within the myocardial region bordering the infarcted tissue (border zone) as early as 24 h post-MI.^46^^,^^47^ Whilst in the early stages after MI the up-regulation of RUNX1 seems specific to the border zone, by 8 weeks post-injury *Runx1* mRNA appears up-regulated in the LV remote from the infarct.^46^ Interestingly, cardiac up-regulation of RUNX1 does not appear to be specific to ischaemic pathology as RUNX1 was also found to be increased experimentally in animal models of: (i) diabetic cardiomyopathy; (ii) pressure overload; and (iii) dilated cardiomyopathy.^51–53^ More recently, RUNX1 has been shown to be involved in zebrafish heart regeneration following cardiac injury.^54^ Cryo-injury resulted in increased RUNX1 expression within various cell types of the myocardium including cardiomyocytes, myofibroblasts, endocardial/endothelial, epicardial, and thrombocytes, where it inhibits heart repair.

## 6. Triggers of RUNX1 expression

Up-regulation of RUNX1 in damaged tissue is reported in non-cardiac tissues including the CNS, lungs, and skeletal muscle.^55–58^ In the nervous system, RUNX1 is up-regulated in response to sciatic nerve crush injury and controlled cortical impact injury.^56^^,^^59^ Comparable with observations made in the heart where RUNX1 was found to be increased in the infarct border zone at 24 h post-MI,^46^ Logan *et al*.^56^ also identified increased RUNX1 expression at the injury site as early as 1-day post-cortical injury. RUNX1 induction at early time points in a range of tissues following different insults may indicate that RUNX1 is up-regulated as part of a common response to injury independent of the precise nature of the original insult.^56^ An interesting comparison can be made between RUNX1 expression in cardiac and skeletal myocytes. Like heart tissue, RUNX1 is increased in skeletal myocytes in a range of pathologies including ischaemic muscle, cardiotoxin treated muscle, and mouse models of Duchenne musclar dystrophy and amylotrophic lateral sclerosis.^57^^,^^60^^,^^61^ Denervation of skeletal muscle is associated with a rapid and marked induction in RUNX1 expression; for example, in rat hindlimb muscle a two-fold induction of *Runx1* mRNA was detected at 1-day post-denervation, rising to 50- to 100-fold by day 5 post-denervation.^58^^,^^62^ Significantly, the up-regulation of RUNX1 in response to denervation can be attenuated by the daily application of either electrical stimulation or stretch to the denervated muscle.^63^ RUNX1 expression is increased in aged muscle alongside denervation and sarcopenia and can similarly be reduced by physical intervention. In a small number of older obese men and women and in aged female mice with sarcopenia resistance training, which is known to preserve the neuromuscular junction and improve innervation,^64^ was found to reduce RUNX1 expression.^65^^,^^66^ Interestingly, in skeletal muscle and in ischaemic cardiomyopathy RUNX1 up-regulation coincides with the up-regulation of the neural cell adhesion molecule (NCAM, CD56).^48^ Whereas, NCAM up-regulation in rhabdomyosarcoma occurred where myocytes were separated by infiltrating tumour, NCAM up-regulation in ischaemic heart tissue arose where cardiomyocytes were separated by scar tissue. If NCAM expression is up-regulated downstream of RUNX1 this may suggest that physical disruption of cell–cell contact or adhesion may contribute to RUNX1 regulation,^48^ possibly by affecting cell communication.

Changes in RUNX1 expression in response to alterations in electrical activity, cell–cell communication or as a result of physical strain or tension may explain why RUNX1 up-regulation is first observed in the border zone following MI. After MI, cardiomyocytes within the border zone exhibit abnormal cell–cell communication due to altered gap-junction distribution and physical disruption of cell contact by interstitial fibrosis and necrotic myocytes, leading to electrical heterogeneity and the precipitation of arrhythmias.^67^ The MI border zone is also subjected to high physical stress and abnormal stretch created at the boundaries between viable cardiomyocytes alongside dead cells and non-contractile scar tissue.^68–70^ As the heart attempts to compensate for the hypocontractile infarct, the remote myocardium also becomes subject to abnormal wall stress which may lead to the up-regulation of RUNX1 in this region at a later stage post-MI.^71^

The apposition of the border zone alongside the infarct means that viable border zone cardiomyocytes are also directly exposed to the inflammatory milieu of the infarcted tissue.^72^ Post-infarction, there is a rapid immune response to cellular damage, resulting in the release of damage signals, chemokines, and cytokines and the recruitment of leucocytes into the damaged area.^73^ Consequently, there is an intense surge of immune signals which could act as a local trigger for RUNX1 expression. Oncostatin-M, which is a member of the IL-6 cytokine family and is secreted by inflammatory cells in response to injury,^74^^,^^75^ has already been identified as a potent inducer of RUNX1 in cultured cardiomyocytes.^47^^,^^51^ In addition, transgenic mice with a heart-specific overexpression of monocyte chemotactic protein-1 (MCP-1) exhibit pathological macrophage infiltration in the myocardium, which occurs alongside enhanced oncostatin-M and RUNX1 expression, providing evidence that an association between inflammation, oncostatin-M, and RUNX1 up-regulation may exist.^47^^,^^76^ Other inflammatory mediators with known links to RUNX1 include the pro-inflammatory cytokine IL-1β and the transcription factor TGF-β. Treatment of a human glioblastoma cell line with IL-1β was shown to up-regulate RUNX1 expression via the p38 MAPK cascade^77^ and TGF-β has been shown to increase RUNX1 expression in a range of cell types including adult hippocampal precursor cells,^78^ mesenchymal stem cells, CD4 T cells, and a mouse hepatocyte cell line.^79^^,^^80^ Currently, the effects of these inflammatory mediators on RUNX1 expression in the myocardium remain unknown.

## 7. RUNX1 and adverse cardiac remodelling

Work conducted using mice with an inducible cardiomyocyte-specific *Runx1* deficiency was the first to provide evidence that the up-regulation of RUNX1 post-MI drives adverse cardiac remodelling.^46^ At baseline, *Runx1*-deficient mice had equivalent echocardiographic contractile parameters to their littermate controls. However, following MI stark differences in contractile function and myocardial remodelling emerged. Whilst classic adverse cardiac remodelling occurred in control mice post-MI including declining systolic function, thinning of the left ventricular free wall, and dilation of the left ventricular chamber, the same parameters were remarkably absent in *Runx1*-deficient mice.^46^ At 8 weeks post-MI, control mice had developed eccentric hypertrophy, characterized by cardiomyocyte elongation and thinning. In contrast, this was notably undetectable in the *Runx1*-deficient mice. The link between RUNX1 and adverse cardiac remodelling has now been corroborated by others in mice^81^ and also in cryo-injured zebrafish hearts, where *Runx1* knockout prevented adverse cardiac remodelling by promoting faster scar degradation.^54^ The cellular composition of the scar was differentially regulated so that smooth muscle and collagen gene expression was significantly reduced, subsequently reducing the amount of collagen, and fibrin deposition. Myofibroblast formation was also reduced whilst fibrinolysis increased, thus allowing invasion of proliferative cardiomyocytes and hence enhancing muscle regeneration.^54^

The improvement in function and myocardial architecture of mouse hearts with *Runx1-*deficiency post-MI may be explained by an effect of RUNX1 on calcium homeostasis in cardiomyocytes. At 2 weeks post-MI, cardiomyocytes isolated from *Runx1*-deficient hearts had increased amplitude of electrically stimulated sarcoplasmic reticulum (SR)-mediated Ca^2+^ release, a faster rate of calcium removal from the cytosol by the SR Ca^2+^-ATPase (SERCA) and a higher SR content.^46^ Although the absolute protein levels of the endogenous SERCA inhibitor phospholamban (PLN) were similar between *Runx1*-deficient and control cardiomyocytes, the proportion of PLN phosphorylated on Ser^16^ and Thr^17^ residues was increased in *Runx1*-deficient cardiomyocytes. This was accompanied by a parallel decrease in protein phosphatase 1, the phosphatase responsible for the dephosphorylation of PLN.^46^ In its phosphorylated state, the inhibitory actions of PLN on SERCA is relieved, allowing enhanced Ca^2+^ uptake into the SR which lowers end-diastolic cytosolic Ca^2+^ concentration and improves the relaxation of the heart during diastole.^82^^,^^83^ SR Ca^2+^ content is also increased, leading to augmented release of Ca^2+^ with each electrically stimulated transient and therefore improved contraction.^84^^,^^85^

The insights offered by McCarroll *et al*., into the protection afforded by *Runx1*-deficiency fit with other studies in the field where improvements in cardiomyocyte calcium handling can have a profound beneficial effect on cardiac structure and function.^84^^,^^86^^,^^87^ Interestingly, the improved contractility noted in *Runx1*-deficient mice occurred in the absence of an effect on infarct size, another key determinant of systolic function after MI.^46^ This finding additionally implies that the protective influence of *Runx1*-deficiency likely relates to the ability of viable cardiomyocytes to functionally compensate for cardiomyocyte death via preserving calcium handling rather than a direct effect of *Runx1*-deficiency on the salvage of ischaemic cardiomyocytes. Importantly and in agreement with mouse studies, several calcium-dependent genes were up-regulated in *Runx1* knockout zebrafish hearts.^54^

Thus far, the direct mechanisms linking RUNX1 to changes in cardiomyocyte Ca^2+^ handling are unknown and require further study. Given the preponderance of Ca^2+^ dysregulation across cardiac pathologies, including diabetic and hypertensive heart disease,^88^^,^^89^ and its contribution to heart failure progression,^90^ it will be useful to establish whether manipulation of RUNX1 in other cardiac disease contexts improve cardiac function.

## 8. Interaction of RUNX1 with signalling pathways involved in cardioprotection and adverse cardiac remodelling

Improved Ca^2+^ homeostasis may not be the exclusive mechanism underlying protective influence of RUNX1 deficiency on the heart. As a master transcription factor, RUNX1 has the potential to simultaneously influence several downstream signalling pathways which may also dictate cardiomyocyte phenotype and the global response of the heart to injury. Although the direct targets of RUNX1 in the heart have not been elucidated, RUNX1 has been demonstrated to interact with signalling pathways in other tissues that are implicated in cardioprotection and adverse cardiac remodelling.

RUNX1 is known to interact with signalling mediated by hypoxia-inducible factor 1-alpha (HIF-1α).^91^ HIF-1α belongs to the HIF family of transcription factors which are key orchestrators of the cellular response to ischaemia.^92^ Many studies have demonstrated that augmented HIF-1α signalling is protective in the context of MI and leads to improved heart contractility, angiogenesis and reduced infarct size^93^^,^^94^; however, chronic activation of HIF-1α signalling in the long term may exacerbate adverse cardiac remodelling and advance progression to heart failure.^95–97^ In haematopoietic cells with forced expression of RUNX1 and HIF-1α, RUNX1 has been shown to physically interact with HIF-1α and reduce transcription of HIF-1α targets including vascular endothelial growth factor (VEGF) and glucose transporter 1 (GLUT 1). Conversely, forced expression of HIF-1α was found to enhance RUNX1-mediated transcription.^98^ In a separate study, RUNX1 overexpression in a glioblastoma cell line down-regulated genes involved in the hypoxic response including the known HIF-1α targets hexokinase 2 (HK 2), caveolin 1 (CAV1), adenosine A2B receptor, and protein phosphatase 1 regulatory subunit 3C gene (PPP1R3C).^99–101^ RUNX1 itself may be up-regulated by HIF-1α, as chemical stabilizers of HIF-1α including dimethyloxalylglycine (DMOG) and cobalt chloride (CoCl_2_) have been shown to increase RUNX1 expression^102–104^ and a correlation between RUNX1 and HIF-1α transcripts was identified in hippocampal transcriptomic data from in-bred mouse strains.^78^ Whether RUNX1 interacts with HIF-1α in the heart has not been elucidated.

TGF-β-mediated signalling is also linked to RUNX1 and implicated in cardiac remodelling. RUNX1 expression is activated by TGF-β and several of the biological effects of TGF-β stimulation have been shown to involve RUNX1 including myofibroblast differentiation and fibrosis.^78^^,^^105^^,^^106^ RUNX1 can direct TGF-β signalling through physical interactions with SMAD proteins, the intracellular transducers of TGF-β receptor activation.^78^^,^^107^ Shortly after MI, TGF-β activation is considered protective and fundamental for infarct healing^108^; however, prolonged and excessive TGF-β signalling promotes adverse cardiac remodelling including interstitial fibrosis and hypertrophy.^108^^,^^109^ Work is needed to determine whether RUNX1 is linked to TGF-β signalling in the heart and whether this interaction is involved in cardiac pathology.

Another means by which RUNX1 can alter cellular function and response to injury is via interaction with microRNAs (miRNAs). miRNAs are small non-coding RNAs which post-transcriptionally modify gene expression by interacting with the 3ʹUTR of target mRNAs. As microRNAs can target hundreds of mRNA sequences and thus simultaneously affect several targets within the one pathway, small changes in their expression has the potential to amount to a significant biological effect.^110^^,^^111^ ChIP-seq data from studies in haematopoietic cells have shown that RUNX1 physically binds over 200 miRNA genes suggesting a potential for RUNX1 to influence a remarkable number of miRNA networks.^112^ An increasing number of studies are providing experimental support for this observation and as a result, the number of validated RUNX1 miRNA targets reported is continuously increasing, including the identification of RUNX1 miRNA targets which have known links to cardiovascular disease.^113^^,^^114^ For example, miR-24 which is down-regulated by RUNX1, decreases in the ischaemic border zone after experimental MI.^114–117^ Moreover, miR-24 appears to have a cardioprotective role within this setting, as local delivery of miR-24 into the border zone after infarction reduced cardiomyocyte apoptosis, decreased infarct size, and improved cardiac contractility.^115^ It is possible that RUNX1 may contribute to reduced miR-24 in the aftermath of MI, and in doing so may adversely affect the cardiac outcome; however, this is yet to be investigated.

The miR-17-92 cluster is a further example of a group of miRNAs that are linked to both RUNX1 and cardiac pathology. The miRNAs in this cluster target the 3ʹUTR of RUNX1 to down-regulate its protein expression and are also themselves targeted by RUNX1 as part of a negative feedback loop.^117^^,^^118^ Of note, the miR-17-92 cluster has been associated with cardiomyocyte differentiation and proliferation, and furthermore, in a mouse model of MI, miR-17-92 overexpression in cardiomyocytes was reported to improve cardiac function and increase the number of proliferating cardiomyocytes within the infarct border zone.^118^^,^^119^

## 9. RUNX1 targets in skeletal muscle

An interesting comparison can be made between the role of RUNX1 in skeletal and cardiac muscle post-injury and may shed light on potential RUNX1 targets in the heart. Both skeletal and cardiac myocytes have a primary contractile function and are thus equipped with highly organized arrangements of myofilaments and complex calcium signalling mechanisms to facilitate this.^120^ Notably, both cell types respond to a range of insults via the up-regulation of RUNX1.^120^ However, in denervated skeletal muscle inactivation of RUNX1 by mutation severely affects the response of muscle fibres to denervation, leading to worsened fibre atrophy, excessive autophagy and structural abnormalities including misalignment and fragmentation of the z discs, dilation of the SR, and an altered myofilament composition.^62^ Thus, in skeletal muscle fibres, RUNX1 appears to protect the structural and functional integrity of skeletal muscle fibres in the face of injury or insult. This contrasts with its role in the heart post-MI, where RUNX1 appears to reduce cardiomyocyte function and promotes adverse cardiac remodelling. The differences between RUNX1 function in cardiac and skeletal muscle are fascinating yet are not easy to reconcile. They perhaps stem from the highly context dependency of RUNX1 function and may arise either because RUNX1 affects distinct processes in the two cell types or may exist because RUNX1 has opposing effects on the same target pathways. Interestingly, several of the 29 genes found to be dysregulated in RUNX1 mutated denervated skeletal muscle have previously been linked to cardiac contractility and cardiac remodelling. For example, RUNX1 was found to maintain or induce the expression of osteopontin, PLN, thrombospondin 1 in the face of denervation.^62^^,^^121^^,^^122^ Following MI, osteopontin up-regulation in cardiomyocytes has been found to negatively correlate with left and right ventricular ejection fraction and positively correlate with cardiomyocyte hypertrophy.^123^ In contrast, thrombospondin 1 up-regulation in the extracellular matrix post-MI appears beneficial by preventing excessive infiltration of macrophages and myofibroblasts into the peri-infarct zone.^124^ Further investigation is now necessary to explore whether RUNX1 acts on similar targets in cardiac muscle and to understand the consequence of these RUNX1 interactions in the context of cardiac disease.

## 10. RUNX1 in differentiation and proliferation

The role of RUNX1 in proliferation and differentiation is well documented. In tissues such as the skin, intestine, mammary gland, and in the haematopoietic system RUNX1 participates in the regulation of stem cell quiescence by directing entry and exit from the cell cycle.^125^^,^^126^ In general, RUNX1 appears to support or maintain proliferation, in part by promoting G1 to S progression through the cell cycle.^126–128^ The control of proliferation appears intimately linked with cellular differentiation, the balance of which may depend on the level RUNX1 expression. Inhibition of RUNX1 in mouse neurosphere cultures with the inhibitor Ro5-3335, a benzodiazepine which may reduce RUNX1 activity by disrupting RUNX1–CBFβ interactions^129^ was shown to inhibit proliferation of neural stem or progenitor cell populations without affecting cell viability or differentiation.^130^ In contrast, lentiviral-mediated RUNX1 overexpression did not affect the proliferative capacity of the cells but instead up-regulated markers of neuronal differentiation and the development of a neuronal-like morphology.^130^

In the heart, RUNX1 may be similarly linked to cell differentiation. This may become important after cardiac injury because the cellular differentiation status of cardiomyocytes in the injured or stressed myocardium^131^ is believed to be closely linked to the remodelling that takes place. Cardiomyocyte dedifferentiation, a phenotype characterized by the loss of the mature sarcomeric structure and the activation of a foetal gene expression profile, occurs in response to injury and has been reported in the ischaemic heart and the MI border zone, in dilated cardiomyopathy, and the pressure overloaded heart.^47^^,^^51^^,^^52^^,^^131–133^ Cells with a dedifferentiated phenotype also stain positive for RUNX1; however, whether up-regulation of RUNX1 plays a direct role in orchestrating dedifferentiation in the heart has not been explored.^47^^,^^51^ Of note, in one study which looked at the ability of adult murine cardiomyocytes to dedifferentiate in an *in vitro* co-culture model with neonatal rat ventricular cardiomyocytes, dedifferentiation, and proliferation of the adult cardiomyocytes coincided with an increase in RUNX1 expression, which was lost when the cardiomyocytes re-differentiated.^132^ Cardiomyocyte dedifferentiation may confer stress resistance to hypoxic conditions and/or metabolic strain which may allow performance to be sustained during pathophysiological stress, providing the damage imposed on the myocardium is self-limiting in nature.^131^ However, in the long term, chronic expression of foetal genes involved in metabolism, calcium homeostasis, and contractility may lead to mitochondrial dysfunction, impaired contractility contributing to adverse cardiac remodelling, and thus promote progression to heart failure.^76^^,^^134^ Improved understanding of the transcription factors driving cardiomyocyte dedifferentiation in response to injury may therefore be a crucial step towards the development of therapies which attenuate adverse cardiac remodelling.

Transcription factors that regulate the balance between proliferation and differentiation are also of interest because of the centrality of these processes to tissue repair and regeneration. In many tissues, resident stem or progenitor cells can proliferate and differentiate to replace injured cells.^135^ RUNX1 has already been identified as a key regulator of stem cell behaviour in the haematopoietic system, skeletal muscle, hair follicles, and the nervous system.^125^^,^^126^^,^^136^ RUNX1 appears to have a role in skeletal muscle regeneration in mice that have lost skeletal muscle dystrophin function (mdx mice).^137^

In the absence of dystrophin, the mice repeatedly undergo cycles of myonecrosis followed by regeneration which preserves muscle mass. However, when the mdx mice were developed to have a skeletal muscle *Runx1*-deficiency in addition to the loss of dystrophin, muscle mass was not maintained beyond 2 weeks, suggesting impaired regeneration of muscle fibres.^137^ The *Runx1-*deficient mdx mice were found to have severe muscle deterioration and fibrosis, accompanied by an impaired exercise capacity and reduced evidence of regenerative fibres. In the same study, primary myoblasts with a RUNX1 deficiency showed reduced proliferation, G1 arrest and premature differentiation whereas, ectopic expression of RUNX1 in myoblasts was found to reduce differentiation.^137^ RUNX1 may facilitate regeneration in this context by preventing premature differentiation of proliferating myoblasts until enough cells have accumulated to permit effective repair.

In contrast to skeletal muscle, the heart has a very limited capacity to regenerate and this is evidenced by the permanent damage to the myocardium caused by infarction.^138^^,^^139^ Although there is evidence suggesting that cardiomyocytes can proliferate, the rate of renewal is very low, estimated at 0.5–2% of cardiomyocytes each year.^140^^,^^141^ Cardiomyocyte proliferation may increase following injury; however, any contributions to tissue repair are considered small.^140^^,^^142^ This contrasts with findings in the neonatal mouse heart as well as zebrafish and newts where a robust proliferative regenerative response is mounted following cardiac resection.^143–145^ Understanding the developmental mechanisms of cardiomyocyte proliferation and regeneration and how these are altered in the adult heart may therefore be a crucial step towards the identification of reparative pathways that could be harnessed to improve cardiac regeneration in the future.^146^ Indeed, a recent study has provided promising evidence that stimulation of endogenous cardiomyocyte proliferation programmes in large mammals is a valid approach to facilitate cardiac repair by increasing myocardial mass and contractility after MI.^147^

Whilst RUNX1 has been identified to be a key player in zebrafish heart regeneration following cardiac injury^54^ its role in cardiomyocyte proliferation programmes in mammals remains unknown. Investigation of this gap in our knowledge is warranted given that RUNX1 regulates proliferation, differentiation, and tissue regeneration in other settings.

RUNX1 has also been shown to interact with the Hippo pathway, a key pathway linked to cardiac regeneration in neonatal as well as adult hearts.^148^^,^^149^ Like RUNX1, the Hippo pathway and its downstream effectors YAP and TAZ are involved in proliferation, differentiation, and expansion of stem cell populations.^150^ Adult mice with a heterozygous deletion of YAP show increased scarring and depressed cardiac contractility following MI and conversely, experimental induction of YAP in cardiomyocytes post-MI has the opposite effect.^151^^,^^152^ RUNX1 has been shown to interact via its PY motif to YAP WW domain to co-regulate target genes.^43^^,^^153^ Co-expression of RUNX1 and YAP leads to the repression of the YAP gene signature and a functional attenuation of the effects of YAP activation in mammary epithelial cell lines.^43^ Although little is known about the interaction of YAP and RUNX1 in the heart, it is interesting to note that oncostatin-M, a potent inducer of RUNX1, is activated downstream of YAP in cardiomyocytes, suggesting that an indirect link between RUNX1 and YAP may exist within the heart.^52^

## 11. RUNX1 in non-myocytes

Whilst studies have focused on the function of RUNX1 within cardiomyocytes to date, a role for RUNX1 in other non-cardiomyocyte cells types may also exist. Other cell types within the myocardium include fibroblasts, endothelial cells, resident immune cells, and neural cells, each with their own role in cardiac physiology and disease. Interestingly, increased *Runx1* mRNA expression is reported in non-cardiomyocyte cells following MI, with 14% of non-cardiomyocytes within the infarct zone expressing RUNX1 at day 1 post-MI and up to 26% and 35% of non-cardiomyocytes expressing RUNX1 within the infarct zone and border zone, respectively, by 2 weeks post-MI.^46^ Similarly, in a mouse model of DCM, RUNX1 expression was increased 4.8-fold in DCM non-myocytes compared to non-myocytes in control hearts.^53^ The exact non-myocyte cell types in which RUNX1 is up-regulated is unknown, and whether this affects cardiac remodelling remains unexplored. However, in a recent study which employed a combination of computational transcription factor-binding analysis and RNA-seq data, the RUNX1 binding motif was identified as a main motif in cardiac fibroblast-specific active enhancers. This suggests that RUNX1 may have an important role in cardiac fibroblasts.^154^ Certainly, RUNX1 has been shown to regulate proliferation in stromal fibroblasts from human prostate-derived mesenchymal stem cells and is involved in the activation of fibroblasts to myofibroblasts.^105^ As the activation and proliferation of fibroblasts in the heart after injury is an integral part of the cardiac repair process and yet in the longer term contributes to cardiac pathology, it will be interesting to see if RUNX1 is implicated within this process in the future.^155^

Many studies document a role for RUNX1 in the development, polarization, and function of the mammalian immune system. A full discussion of RUNX1 in inflammation and immunity is beyond the remit of this review and can be found elsewhere.^156–158^ However, it is important to consider that the ability of RUNX1 to modulate the inflammatory phenotype in response to damage may have profound implications in the injured heart, where immune cell function impacts upon remodelling and repair.^159^^,^^160^ Generally, a loss of RUNX1 function is associated with heightened inflammatory responses.^161–163^ Mice with a *Runx1* deletion in alveolar epithelial cells had up-regulated NF-κB signalling, augmented pulmonary inflammation and an earlier onset of death after pulmonary LPS exposure.^55^ Further studies have suggested that RUNX1 attenuates NF-κB signalling by directly binding IκB kinase (IKK) in the cytoplasm and preventing it from targeting IκBα, an endogenous inhibitor of NF-κB, for degradation.^55^^,^^162^ However, common to other RUNX1 functions, the effect of RUNX1 on inflammation appears context dependent, as in some settings RUNX1 appears pro-inflammatory in its action. For example, in myometrial cells silencing of *Runx1* with siRNA led to reduced up-regulation of IL-1β, IL-6, and the adhesion molecules VCAM1 and ICAM1 mRNA in response to treatment with TNF-α.^164^ Similarly, in mouse peritoneal macrophages cultured *in vitro* the C-terminus of RUNX1 interacts with the p50 subunit of NF-κB to co-transcriptionally activate the production of IL-6 in response to stimulation with LPS.^165^ The findings that RUNX1 can act as both a positive and negative regulator of NF-κB signalling is particularly intriguing, as NF-κB is a major orchestrator of the inflammatory actions of cytokines in heart disease.^166^ Importantly, NF-κB has been identified as both a cardioprotective mediator and an active participant in the progression of cardiac disease depending on the spatial-temporal resolution of its activation.^166^^,^^167^ Therefore, any factor capable of modulating its activity or directing its transcriptional profile in either a positive or negative manner may be expected to influence the outcomes of cardiac pathology.

Like the other non-myocyte cell types resident in the heart, endothelial cells are gaining increasing attention for playing an active role in cardiomyocyte function and disease progression.^168–170^ Whilst a comprehensive review of the functions of RUNX1 in endothelial cells is beyond the scope of this review, the link between RUNX1 and angiogenesis will be considered further due to the significance of angiogenesis in tissue salvage post-MI and in cardiac remodelling.^171^ The importance of RUNX1 in the developing vasculature is highlighted by the phenotype of *Runx1*-deficient embryos which exhibit defective vascular formation and suffer fatal haemorrhages in the CNS, pericardium, and peritoneum.^9^^,^^37^ Generally, RUNX1 has a pro-angiogenic effect on endothelial cells. In endothelial precursor cells derived from mouse embryos, RUNX1 was shown to direct angiogenesis by inducing endothelial cell differentiation and vascular network formation through the induction of VE-cadherin and repression of the anti-angiogenic factor insulin-like growth factor-binding protein 3.^172^ Similarly, siRNA knockdown of *Runx1* in cultured human retinal microvasculature endothelial cells (HRMECs) decreased endothelial migration and proliferation.^173^ In endothelial tube formation assays, an *in vitro* methodology used to study the angiogenic capacity of cultured cells, treatment of HRMECs with the RUNX1 inhibitor Ro5-3335 was associated with reduced tubular structure formation, indicating depressed angiogenic function.^173^ Interestingly, in another study also using tube forming assays, treatment of human umbilical vein endothelial cells (HUVECs) with conditioned media prepared from RUNX1-silenced glioblastoma cells was able to have a similar suppressive effect on angiogenesis.^77^ This suggests that RUNX1 expression in non-vascular cell types may be able to direct endothelial function and angiogenic capacity via the release of paracrine signals.

Paradoxically, RUNX1 has been shown to both directly and indirectly limit the expression of the potent pro-angiogenic factor VEGF.^98^^,^^174^^,^^175^ This would suggest that RUNX1 may limit angiogenesis in some circumstances, and in support, silencing of RUNX1 in acute myeloid leukaemia cells increased VEGFA promoter activity and conditioned media from these cells led to a significant improvement in the *in vitro* angiogenic capacity of HUVECs.^175^ Like its other functions, it may be that RUNX1 acts in a context-dependent manner and possess the capacity to both stimulate or restrain angiogenic potential depending on the cellular milieu.

In the injured heart, expansion of the vascular network through angiogenesis is a key response which protects against adverse remodelling and progression to heart failure.^176^^,^^177^ In view of the evidence linking RUNX1 to angiogenesis, it may be valuable to explore whether the up-regulation of RUNX1 in cardiomyocytes or in non-myocytes in the border zone following MI influences angiogenesis within this region.

## 12. RUNX1 as a novel target in heart disease

Heart failure remains a major global health and economic burden.^178^ There is a persistent unmet need for better treatments as patients with heart failure with reduced ejection fraction continue to have poor long-term outcomes. Importantly, there appears to be a lack of translation between basic science discovery and drug development with most therapies failing phase III clinical trials despite promising phase II testing.^179^ Critically, there are currently no approved treatments for heart failure patients with preserved ejection fraction, although phase III trials are on-going (Novartis PARAGON-HF trial and AstraZeneca DETERMINE trial).^180^

Novel therapies aimed at targeting a master-regulator transcription factor such as RUNX1 may achieve a more efficacious therapeutic response by impacting on multiple downstream signalling pathways to mitigate the adverse cardiac remodelling that initiates heart failure. Given its involvement in systolic dysfunction^46^ inflammation,^164^^,^^165^ fibrosis and angiogenesis,^172^^,^^173^ RUNX1 has been highlighted as an attractive therapeutic target.

Transcription factors have historically been viewed as unlikely and challenging ‘druggable’ targets due to their pleiotropic actions in multiple cell types and systems during normal development and disease.^181^ More recently and thanks to advances in our knowledge of transcription factor structure and function, transcription factor drivers of cancer have been directly targeted using small molecule protein–protein interaction inhibitors.^182^

Importantly, the small molecule benzodiazepine Ro5-3335 was identified as a disruptor of the CBFβ–RUNX interaction.^129^ Ro5-3335 was shown to repress CBFβ/RUNX-dependent transactivation in reporter assays and repress RUNX1-dependent haematopoiesis in zebrafish by altering the conformation of the CBFβ–RUNX1 complex. Subsequently, Ro5-3335 has been used to inhibit RUNX1 during a variety of pathophysiological processes including, but not exclusively in, macrophages during septic shock,^165^ in retinal angiogenesis^173^ and, acute myeloid leukaemia.^183^ However, direct binding of Ro5-3335 to CBFβ or RUNX has not been well documented, thus whether Ro5-3335 is a direct inhibitor of Runx remains contentious.^184^

Illendula *et al.*^184^ developed a selective small-molecule inhibitor of RUNX activity. Further optimization of this compound led to a new small-molecule inhibitor, AI-14-91, which binds selectively to CBFβ and prevents it from binding RUNX proteins via an allosteric mechanism.^185^ AI-14-91 has been optimized and shown to inhibit RUNX1 function in leukaemia and basal-like breast cancer cells.^185^ As mentioned, adverse cardiac remodelling is a complex response to injury involving cardiomyocyte loss, hypertrophy, fibrosis, extracellular matrix remodelling, and angiogenesis. As RUNX1 has the potential to impact all these processes, it is pertinent to determine whether AI-14-91 can prevent adverse remodelling and progression to heart failure following cardiac injury and is the focus of on-going studies.

## 13. Conclusions

Accumulating evidence supports that RUNX1 is up-regulated at early time points in the heart in response to a range of cardiac pathologies. New developments in the field have demonstrated that RUNX1 activation in cardiomyocytes after MI is detrimental to systolic function and cardiac structure and have thus unveiled RUNX1 as an attractive novel target to mitigate adverse cardiac remodelling. Understanding of the role of RUNX1 in the heart is in its infancy and there are many pertinent questions to be asked about the identity of the downstream targets of RUNX1 in the heart. Crossing discipline boundaries to understand RUNX1 biology across different organs has offered insight into the potential functions of RUNX1, however, due to the strong context-dependent nature of RUNX1 further work is now needed to evaluate whether the effects of RUNX1 observed in other tissues translate into the cardiac context. This exploration should extend beyond cardiomyocytes to study the role of RUNX1 in other cardiac cell types in heart physiology and pathology. As a master transcription factor, RUNX1 has the potential to act across an entire network of signalling pathways. Unravelling the functional consequences of this network represents an exciting challenge with a translational potential.

**Conflict of interest:** none declared.

## Funding

Funding was received from the British Heart Foundation, PhD studentship FS/15/64/32035 and Project grant PG/18/9/33548.
